# Achieving a Covid-19 Free Country: Citizens Preventive Measures and Communication Pathways

**DOI:** 10.3390/ijerph17134633

**Published:** 2020-06-27

**Authors:** Irene (Eirini) Kamenidou, Aikaterini Stavrianea, Christina Liava

**Affiliations:** 1 Department of Management Science and Technology, International Hellenic University, Kavala Campus, 654 04 Agios Loukas Kavala, Greece; 2 Department of Communication and Media Studies, National and Kapodistrian University of Athens, 105 62 Athens, Greece; aikstavria@media.uoa.gr; 3 4th Department of Internal Medicine, Aristotle University of Thessaloniki, Hippokration General Hospital, Konstantinoupoleos Str.49, 546 42 Thessaloniki, Greece; christinaliava@aol.com

**Keywords:** COVID-19, SARS-CoV-2, preventive behavior, research, segmentation, marketing communication

## Abstract

The Coronavirus Disease 2019 (COVID-19) has spread on a global scale in an extremely short time, causing hundreds of thousands of deaths, and, at the same time, triggering extreme panic. Prevention in medicine is considered the best protection action for individuals in order to avoid infections. This study investigates whether Greek citizens (*N* = 3359) take the necessary precautions to prevent developing the COVID-19 disease, and it segments them based on homogenous behavior groups. Lastly, it provides communication techniques that should be implemented, targeting each citizen segment for a long-term COVID-19 free country. Data analysis revealed the extent of the applied precaution measures. The ones most applied by citizens were to avoid non-mandatory transportation, contact with individuals with respiratory symptoms, and individuals of high risk for severe illness (vulnerable groups). On the other hand, the least applied measures are daily checks of body temperature, monitoring for fever, cough, or dyspnea, use of a face mask when in public places, or when using public transportation. Additionally, cluster analysis revealed five groups of citizens based on self-reported behavior, namely, the Meticulous Proactive Citizens, the Self-isolated Citizens, the Cautious Citizens, the Occasionally Cautious Citizens, and the Unconcerned Citizens. Communication strategies targeting each segment are also discussed.

## 1. Introduction

The most recent threat to the global community is the ongoing outbreak of the disease known as Coronavirus Disease 2019 (COVID-19) [1]. Initially, COVID-19 was first reported in December 2019 in Wuhan, the capital city of Hubei province in China as pneumonia cases of unknown etiology [2]. On January 7, this novel coronavirus was officially identified by the Chinese Center for Disease Control and Prevention (CDC). This virus is responsible for the disease COVID-19 and is structurally similar to SARS [3]. The newly identified virus was named “severe acute respiratory syndrome coronavirus 2” (SARS-CoV-2) because it affects the respiratory system and causes severe acute respiratory syndrome (SARS) [1]. Coronaviruses belong to a family of viruses that cause illnesses like the common flu, severe acute respiratory syndrome (SARS), and the Middle East respiratory syndrome (MERS) [4,5]. It should be noted that, in the past 18 years, many instances of public health emergencies have been triggered by viruses, such as the SARS-CoV disease in 2002–2003, the Middle East respiratory syndrome (MERS-CoV) in 2012 until now, and the Ebola virus disease (EVD) from 2014–2016 [1,6,7,8,9,10].

A month later (January 2020), the World Health Organization (WHO) publicly announced it is considered a public health emergency that required global attention [11]. Rapidly, the disease spread from Wuhan to other areas worldwide and developed into a pandemic in March 2020. However, the COVID-19 outbreak has caused severe consequences to the global public health, medical communities, and to the socioeconomic status of a considerable number of countries [1]. The COVID-19 disease is associated with substantial morbidity and mortality while 8,802,328 confirmed cases and 464,620 deaths have been reported as of 21 June 2020 (12:22 Greek time), according to the COVID-19 Dashboard by the Center for Systems Science and Engineering (CSSE) of the John Hopkins University [12]. The usual symptoms of the disease are respiratory, which indicates droplet transmission [13]. However, case studies have reported that some patients with SARS-CoV-2 infection have titers of virus in feces, which suggests fecal-oral transmission [14,15,16]. Signs and symptoms of Covid-19 disease may appear from 2 to 14 days after exposure, whereas 97.5% of the patients develop symptoms within approximately 11.5 days after infection [13,17,18,19]. The most frequent clinical manifestations include fever, cough, dyspnea, myalgia, and fatigue. Some patients also develop gastrointestinal symptoms such as anorexia, nausea, and diarrhea [2,9,13,17,20,21]. The severity of Covid-19 symptoms can range from very mild to severe [13], while some people may show no symptoms at all (i.e., they are asymptomatic) [22]. Transmission of SARS-CoV-2 occurs predominantly after days of illness and is associated with moderate viral loads in the respiratory tract early in the disease with viral loads reaching the highest point approximately 10 days after symptom onset [23]. Among the risk factors associated with severe COVID-19 disease, one encounters older age (>65 years), cardiovascular disease, chronic lung disease, hypertension, diabetes, and obesity [13,18,21,24].

Droplets are typically released after coughing or sneezing and they usually fall within a few meters. The possibility of virus transmission decreases if people maintain a distance of at least 2 m [25,26]. SARS-CoV-2 may remain for days on cardboard, plastic, and stainless-steel materials, which may also play a role in its transmission [27]. One major challenge towards preventing the spread of SARS-CoV-2 is that pre-symptomatic people are infectious. Contemporary studies indicate that patients may be infectious 1 to 3 days before symptom onset. Additionally, up to 40–50% of cases may be attributed to transmission from asymptomatic or pre-symptomatic people [28]. Prior to or soon after symptom onset, patients reveal high nasopharyngeal viral levels that fall during the following week [29]. Patients with severe disease could spread the virus for a longer time, even though the extent of infectious viral transmission is uncertain [30].

The relevant literature emphasizes that citizens should take precautionary measures to protect themselves from infection or spreading the virus, and it discusses various practices that should be implemented [31,32,33]. Public health intervention tactics include countries imposing quarantine, case detection, and isolation [25,32,34,35,36,37].

Although the COVID-19 disease is an ongoing situation crisis, literature published is of extreme importance to obtain insights from different stakeholders, i.e., government officers, governmental organizations, health care professionals, and citizens. While further research is needed in diverse aspects, associated with the COVID-19 crisis, protective prevention tactics will not flourish if citizens do not want to comply with them. Thus, citizens’ approach toward prevention tactics against COVID-19 is extremely essential for any long-term results retrieved. Therefore, the following research questions emerged from the relevant literature and the importance of the public reaction.

RQ1: What precautions do citizens take to protect themselves from being infected from the SARS-CoV-2 virus and spread the COVID-19 disease?

RQ2: Can citizens be grouped based on their behavior regarding self-implemented protective actions?

RQ3: What profile does each of these groups of citizens exhibit?

RQ4: What communication practices should be enforced for each citizen group to raise awareness and motivation in order for them to comply with the preventive actions against the SARS-CoV-2 virus for long-term results?

From the above research questions, the following aim and objectives were drawn. The aim of the study is to explore the tactics used by citizens for preventive reasons regarding SARS-CoV-2 virus and against spreading the COVID-19 disease (it answers RQ1). Additionally, its objectives include (1) to group participants based on similarities in their behavior (it answers RQ2), (2) to create the profile of the derived segments (it answers RQ3), and (3) to suggest marketing communication techniques, which will not only raise awareness but remind people of the preventive actions that should be taken to lead to a COVID-19-free country (answers RQ4).

Research published referring to the COVID-19 disease is continuously increasing since the crisis developed is exceptionally severe, and a vaccine or drug that could cure it has not been discovered yet [38]. Hence, this research contributes to the academic work in the following ways.

1. It provides insights from a country that has managed to have a few deaths due to the COVID-19 disease. 

2. It studies actual self-reported tactics of prevention from the COVID-19 disease, which, at present, is an understudied issue (few peer-reviewed academic articles exist to our knowledge). 

3. It provides an in-depth understanding of research referring to citizens’ behavior that is not connected to the health sector in any way.

4. It provides an in-depth knowledge of citizens’ behavior in crisis situations.

5. It segments citizens based on their actual self-reported preventive behavior, which is an issue that, to the best of our knowledge, has not been investigated yet.

With respect to context, this paper exploits data from Greece, which is a European country that has managed to have a relatively small number of confirmed cases and deaths related to the COVID-19 disease. In Greece, the first confirmed case of COVID-19 was detected on 26 February 2020, while other suspicious cases of COVID-19 arose from people returning from Italy [39]. The day before (25 February 2020), the government announced that it issued a legislative act (No. 42/25–2–2020) for protective reasons. The Legislative Act included articles and covered issues such as imposing precautionary controls on ports, airports, and railway stations, especially where there is a connection with high-risk countries. Similar measures also followed for public gathering places, such as schools, churches, the suspension of operation for all educational units, measures to restrict the movement of means of transport within the territory, and temporary home restraint [40]. These measures started one-by-one to be applied during the following days and weeks. On March 10, the closure of all educational institutions of all levels in Greece was announced, and distance learning was implemented. On 13 March 2020, business activities were suspended throughout the country, apart from food retailers and pharmacies. On March 14, the first spot “I am closing the door to COVID-19” appeared in traditional and digital media [41]. The measures for transportation were implemented and flights from and to Italy were banned. On March 22, the joint decision of the Ministers of Civil Protection, Health, and Interior no. Δ1α/Γ.Π.οικ.20036/22.3.2020 (Β ‘986) regarding the commuting of citizens concerning the entire Greek territory without any exceptions was issued whereas movement was feasible under specific circumstances and only with a cellular phone text-short message service (SMS) or written permission. Moreover, penalties for violating the rules of temporary traffic restrictions were imposed, which included fines and even imprisonment in some extreme cases [42,43,44]. On March 23, all flight air connections with the United Kingdom were paused, and the Greek Ministry of Foreign Affairs in Turkey also announced that all connections (air, road, rail) with Turkey are cutoff as well [45]. On March 26, Aegean airlines suspended all its flights to and from abroad while other flights to and from Greece were also suspended [46]. These measures were maintained up to 3 May 2020. The total number of COVID-19 cases reported on the 3rd of May were 2626, while 144 deaths due to COVID-19 were confirmed [47]. The same source states that, out of the total 2626 cases, 595 (22.7%) are considered travel-related from abroad. Lastly, it reveals that 1303 (49.6%) cases are associated with an already known case, and the rest are neither related to traveling nor to another known case or they are still under investigation [47]. From May 4, the restrictions mentioned above were modified, and civilians could go out without an SMS or printed certificate. Additionally, some of the businesses started to function while transportation to another prefecture was allowed from May 18 onward. The measures will be reevaluated in the future [48]. 

## 2. Materials and Methods 

### 2.1. Research Design

Within the above theoretical framework, this research explores 3359 Greek citizens’ preventive behavior from the COVID-19 disease. Data were collected utilizing a questionnaire developed specifically for this reason based on previous studies, see, for example, [25,32,34,35,36,37,49,50].

A small-scale pilot test (*N* = 140) led to minor modifications regarding syntax and grammar by assisting researchers to finalize the questionnaire for the actual field research and to ensure face validity. A combined non-probability sampling method was applied (convenience, snowball, and criteria) mainly via online platforms. The main selection criteria that this research posed was that participants should not be employed or be students in the health care sector. Thus, subjects that were employed in the healthcare sector in any way (i.e., health care providers or employees), or who were students (undergraduate, graduate, and postgraduate) in healthcare schools or departments were excluded from the study. Additionally, subjects that did not offer their consent for their questionnaire answers to be used for analysis were also excluded. For the younger subjects, data collection was carried out with an online questionnaire. For the older people’s responses or people that did not have access to the Internet, students that were trained prior to the study were recruited. These students conducted personal and phone interviews with their relatives and acquaintances being rewarded with an extra credit on their grade. The online data collection link remained active from March 1 to 13 May 2020. This procedure led to ensuring a sample of 3359 valid questionnaires, which is considered appropriate for the study’s aim and objectives as well as for the statistical analysis realized.

Ethical approval: “There are no ethical issues involved in the processing of the questionnaire data used in the study. The necessary consents have been obtained by the persons involved, and the anonymity of the participants has been secured. All procedures performed in studies involving human participants were in accordance with the ethical standards of the International Hellenic University Research Committee and with the 1964 Helsinki declaration and its later amendments or comparable ethical standards”. Permission was obtained under the No. 2/20.1.2020 decision of the Coordinating Committee.

### 2.2. Measures

The issue that is introduced and thoroughly examined in this research is closely interwoven with the precautions taken by citizens to prevent infection by the SARS-CoV-2 virus and its potential spread. This topic was examined via a one multi-item question: “Please state how often you take the following precautions to protect yourself and avoid the spread of the SARS-CoV-2 virus”. This question incorporated 27 items-statements of preventive actions, which were presented on a seven-point Likert-type scale (1 = Never, 2 = Very rarely, 3 = Rarely, 4 = Neither rarely/nor frequently (sometimes/occasionally), 5 = Frequently, 6 = Very frequently, and 7 = Always). The above items were adopted from the studies performed by various organizations and authors [25,32,34,35,36,37,49,50,51].

Data was analyzed with the IBM SPSS ver. 24 statistical package. Data analysis covers descriptive statistics, i.e., frequencies, percentages (%), and mean scores (addressing RQ1 and the overall aim of the study). Additionally, cluster analysis was performed to group citizens based on their self-reported proactive behavior while ANOVA tests were run to confirm that each cluster is different from the rest (answers RQ2/objective No. 1). Lastly, chi-square tests were performed to examine participants’ socioeconomic and demographic characteristics in an attempt to explore each segment’s profile (it answers RQ3/objective No. 2).

The reliability and validity of the multi-item questionnaire analyzed in this paper were also assessed. Specifically, to ensure the content validity of the items, these were adopted from the World Health Organization, Governmental Organizations, and the above-mentioned peer-reviewed academic published papers. The questionnaire’s face validity was confirmed by the pilot test where an additional question was added regarding readability and understanding [52,53]. The reliability of scale with Cronbach alpha was calculated to confirm the internal consistency of the scale, which produced Cronbach a = 0.962. This is regarded as acceptable [54].

## 3. Results

### 3.1. Sample Profile

Females subjects were slightly overrepresented (Table 1). In relation to age, the group 36–45 is underrepresented, while the <25 age group is overrepresented. About one-half of the participants were married and they possessed at least a bachelor’s degree. Likewise, two professional groups stood out: the dependent group (i.e., housekeepers, students, and unemployed) and the employee group (both from the private and civil sector). Moreover, two-thirds of the sample reside in an urban area. Lastly, the majority of the participants have a net income ≤of 1000.00€ per month.

### 3.2. Self—Reported Prevention Behavior

Participants were called to report their proactive protection behavior against the COVID–19 disease and against increasing the probability of spreading the SARS–CoV–2 virus (RQ1/main aim of the study). Proactive behaviour was rated on a 7–point Likert type scale (1 = Never, 2 = Very rarely, 3 = Rarely, 4 = Neither rarely/nor frequently (sometimes/occasionally), 5 = Frequently, 6 = Very frequently, and 7 = Always). Table 2 presents participants’ answers in percentages and mean scores (MS) in descending order. Table 2 reveals that, overall, participants do comply with the suggested and restrictive actions of the WHO, governments, and academics. The three most used protective measures are: “Avoid all non–mandatory transportation and travel” (MS = 6.22), “Avoid contact with individuals who have respiratory symptoms” (MS = 6.21), and “Avoid contact with individuals at high risk for severe illness (vulnerable groups)” (MS = 6.18). On the other hand, the least frequently applied measures by citizens are “Check daily body temperature, monitoring for fever, cough, or dyspnea” (MS = 4.11), “Use face mask when in public” (MS = 4.69), and "Use a mask when using public transportation" (MS = 4.86).

### 3.3. Cluster Analysis—Citizen Segmentation Based on Preventive Behavior

Cluster analysis was performed in order to group citizens into homogenous groups with similar behavior (RQ2/objective No. 1). Previous literature suggests that audience segmentation in the healthcare setting is significant and efficient [55,56,57]. Moreover, literature provides examples of the first conducting factor analysis to decrease the number of items in fewer variables, and then execute cluster analysis based on these decreased variables [58,59].

However, Dolnicar and Grün [60,61] as well as Dolnicar & Lazarevski [62] claim that it is better to directly perform cluster analysis since all the information of the data obtained is contained, and detailed information is gained. Since this multi–item question concerns proactive protective measures from COVID–19, it was considered that in–depth knowledge is needed of citizens’ behavior. Thus, K means Cluster Analysis based on the 27 items was performed, providing five segments having a practical and physical interpretation [63,64]. The criteria for the validity of the solution of K Means Cluster Analysis proposed was adopted from Kamenidou et al. [65] by implementing in the analysis the steps applied. More precisely, initially, hierarchical cluster analysis was applied in order to identify a range of solutions as well as to gain a first estimation of their centroids. The second step was to compare this solution with other solutions coming from randomly selected data subsets [66]. The third step was to compare this solution with other solutions originating from applying fewer variables [67]. Lastly, the fourth stage was to examine solutions with a different number of clusters. Taking into consideration the above steps/criteria as well as that the final clusters should receive practical and physical meaning [63,64], the five–cluster solution was selected as the most appropriate one. Additionally, the ANOVA test results revealed that all items contributed to differentiating the five clusters [68] whereas a larger F value means greater separation between clusters [69]. It is noted that *p* < 0.001 for all comparisons. For each cluster, the final cluster centers (FCC) are calculated as the mean of every variable within each final cluster and it reveals the characteristics or behavior of the typical subject–case for each cluster [70]. Table 3 presents the five segments based on self–reported preventive behavior, the final cluster centers (FCC), the number of participants per cluster (N), and the ANOVA tests (F values). 

Clusters were named after subjects’ distinctive primary proactive behavior regarding COVID–19 disease. Thus, the first cluster is labeled the “Meticulous Proactive Citizens”, the second is named the “Self–isolated Citizens”, and the third group is labeled the “Cautious Citizens”. Moreover, the fourth group is named “Occasionally Cautious Citizens” and the last segment carries the label the “Unconcerned Citizens”.

Additionally, chi–square tests were performed between cluster–segments and citizens’ socioeconomic and demographic characteristics to explore any statistical differences (Table 4) and profile in–depth the segments [65,67].

### 3.4. Cluster Profile

Table 5 presents the profile of each segment of citizens based on their proactive protective behavior (RQ3/objective No. 2). Literature suggests that profiling of clusters based on different variables (such as demographics, psychographics, and behavioral) are particularly significant to the maximum effective communication impact since communication varies depending on the targeted segment [55,71,72].

Through a combined observation of Table 3 and Table 5, the segments’ profile has as follows: Cluster I: “*Meticulous Proactive Citizens*.” This cluster consists of 1634 citizens representing 48.6% of the total sample, which constitutes the largest group of the produced segments. This segment has the highest centroids compared to the other segments in all cases with almost all FCC > 6.0 (i.e., very frequently tending to always), excluding two cases (with FCC = 5.90 and 5.21, respectively). Since the citizens of this group take all the necessary precaution measures, this group is labeled the “Meticulous Proactive Citizens”. As to their socioeconomic and demographic profile (Table 5), in this cluster, females outweigh male participants, while, in terms of age dispersion, excluding the age group of <25 and 55–65 (20.2% and 19.5% respectively), ages are almost equally represented. This group, though, has the highest percentages of participants who are 66+ years of age (elderly). These are people that belong to a vulnerable group. It also has the highest percentage of the 55–65 age category. Additionally, the subjects of this segment are mostly married. This group has the largest percentage of children in the household compared to the other groups, and it also has the highest percentage of divorcees. The citizens in this group are highly educated with 46.2% holding at least a bachelor’s degree. However, this is the segment with the largest percentage of low education citizens with 11.9% having attended only elementary school, while another 3.9% could be considered uneducated (they have not completed primary education studies). As to their profession, this group incorporates the highest percentage of employees (federal and private) and pensioners compared to the other segments and a low percent of dependents in comparison with the groups No. 2, 4, and 5. Regarding the area of residence and personal net monthly income, the majority reside in an urban area and belong to the 600.01–1000.00€ category. Combined age, marital status, and children in the household lead to the assumption that grandparents (55+) take care of their grandchildren. Thus, they need to be safe in order for their grandchildren to be safe as well. This is an assumption also substantiated by the fact that they are meticulous in their preventive behavior.

Cluster II: “*Self-isolated Citizens*.” This cluster consists of 599 citizens representing 17.8% of the total sample. This segment has 19 FCC > 5.70. In other words, they tend to frequently take the specific proactive measures in order to protect themselves from COVID–19. Additionally, three items have FCC < 5.50 and >3.51, which means that, occasionally, they take these specific proactive actions for self–protection, and five items have FCC > 2.00 and ≤3.50. Thus, this segment can safely be labeled as the “Self–isolated Citizens” because, judging from their behavior, it seems that they prefer self–isolation rather than going to public areas. Therefore, this minimizes exposure to the SARS–CoV–2 virus. Due to self–isolation and abstaining from public places, proactive measures regarding circulation in public areas are not needed. This cluster is overrepresented by females (59.3%) of age 46–65 years old (41.2%), single (48.1%), and with no children in the household (65.1%). Additionally, this group is highly educated (59.1% has at least a bachelor’s degree). With respect to their profession, 43.2% have either retired or are dependent on others. Regarding the area of residence, it is the group with the highest percentage of citizens residing in urban areas (64.9%), which justifies their isolation, since urban areas had more COVID–19 cases than rural ones. As to personal net income, excluding the category of 2000.01€+, the other categories are all almost equally represented.

Cluster III: “*Cautious Citizens*.” This cluster consists of 636 citizens representing 18.9% of the total sample. This segment has 6.00 ≤ FCC < 4.00. Specifically, 25 items have FCC ≥ 5.00 and <6.00, and two items have 4.00 < FCC ≤ 4.50, suggesting that they frequently comply with the proactive measures to protect themselves from COVID–19. The participants in this segment do understand that they should comply with the measures to protect themselves but are not meticulous about applying them. Thus, this segment can safely be labeled as “Cautious Citizens”. This group of citizens is overrepresented by males (55.3%), and the age groups 36–45 and 66+ have almost equal percentages (12.6% and 12.1%, respectively). Additionally, the rest of the age categories are also somehow equally represented. Moreover, they are basically married (51.1%), and come with no children (<18 years of age) in the household (67.3%). Furthermore, this segment comprises mostly of citizens with a bachelor’s degree (39.8%) and secondary education (27.2%). As to their profession, this group compared to the other segments entails the lowest percent of dependents (28.8%), and the highest percentage of private employees (23.7%), which resides in their majority in urban areas (59.1%). Lastly, two categories stand out as to personal net monthly income: the 350.01–600.00€ (26.7%) and the 600.01–1000.00€ (26.3%) category.

Cluster IV: “*Occasionally Cautious Citizens*.” This cluster consists of 338 citizens representing 10.1% of the total sample, which forms the second smallest group of citizens. This segment has 19 FCC < 5.00 and >3.70, which signifies that they tend to occasionally (or sometimes) take proactive measures in order to protect themselves from COVID–19. Additionally, only two items have FCC ≥ 5.00 (frequently take these specific proactive self–protection actions). Lastly, six items have 2.00 < FCC ≤ 3.50. Thus, this segment can safely be labeled as the “Occasionally Cautious Citizens” since, generally, they seem to sometimes engage in the above proactive measures. This cluster is clearly overrepresented by males (60.7%), young participants (47.6% have aged up to 35 years old), single (50.9%), and with no children (<18 years old) in their household (69.1%). The majority of the sample has completed secondary and postsecondary education (42.3%) and a bachelor’s degree (35.8%). As to profession, this group incorporates the highest percentage of businesspeople (18.9%) compared to the other four segments. Most of them reside in urban areas, while two categories stand out in relation to their personal net income: those that have income <350.00€ per month (27.2%) and the 600.01–1000.00€ per month (27.2%). Even though this group consists mainly of urban residents, it has–compared to the other segments–the second highest percentage of residents living in rural areas (44.1%), partially explaining their occasional proactive behavior. Since transportation restraints exist, and people in small towns and villages have access to a small circle of people, they know whom and what each person encountered, and, thus, they do not need to be meticulous in their prevention behavior.

Cluster V: “*Unconcerned Citizens*.” This cluster consists of 152 citizens representing 4.6% of the total sample and they are the smallest group. This segment has all FCC < 3.00 and >2.00, which means that they rarely take proactive measures to protect themselves from COVID–19. Consequently, this segment could be safely labeled as the “Unconcerned Citizens.” The demographics of this reveal that it is clearly overrepresented by males (61.2%), young participants (37.9% have aged up to 25 years old), single (52.6%), and with no children (<18 years of age) in the household (71.5%). The majority of the people in this group have received secondary and post–secondary education (39.9%) and they hold a bachelor’s degree (36.2%). As to profession, this group incorporates the highest percentage of dependents (40.1%) in comparison with the other groups. With respect to their area of residence, the majority reside in an urban region. At the same time, this segment also has the highest percentage of people residing in rural areas compared to other segments, which justifies, up to a point (transportation limitation, access to a small circle of people, knowledge of fellow citizens’ contacts), the reasons for “taking loosely” the proactivity by the government measures. Regarding monthly personal net income, the people in this segment are considered as a high–income people with 38.2% having a personal net monthly income >1000.00€/month. At the same time, it encompasses the highest percentage of participants with net personal monthly income exceeding 2000.00 € per month.

## 4. Discussion

### 4.1. Addressing RQ1 (Main Aim of the Study)

This research is in line with previous research findings that explored citizens’ practices during the COVID–19 outbreak [36,73,74,75]. Specifically, Zhong et al. [36] studied citizens’ practices during the outbreak of COVID–19 in Hubei, China (*N* = 6910), and found that citizens avoided crowded places (96.4%), wore masks when going out (98.0%), washed their hands after touching objects and surfaces in public places (78.9%), and used a hand sanitizer, which contained at least 60% alcohol (81.2%). Additionally, social distancing and not touching the face was practiced by citizens, at least, very frequently (by 69.4% and 71.7% of the sample, respectively) while concerning respiratory etiquette and self–isolation (i.e., avoiding any contact with other people) was always practiced by 49.2% and 43.2%, respectively. On the contrary, 60.5% applied this practice to people coming from abroad. In their research (Japan, *N* = 2400), Machida et al. [73] identified how often the citizens implemented the personal protective measures suggested by the World Health Organization (namely hand hygiene, social distancing, not touching the eyes, nose, and mouth, respiratory etiquette, and self–isolation). They found that the prevalence of the above five personal protective measures ranged from 59.8% to 83.8% with the lowest being avoiding touching their eyes, nose, and mouth. Chen et al. [74] researched prevention and control behavior in Anhui Province (*N* = 4016), and concluded that almost all participants avoided gatherings and seldom went out of the house (97.4%). They also wore masks (93.6%), and avoided crowded and closed places (91.5%). Muto et al. [75] in their study, focusing on Japanese citizens (*N* = 11,342), revealed that 85% and 86% of the participants practiced social distancing and frequent hand washing, as advised by their government. Compared to the above studies, the present research found that the most applied protective measures are avoiding: transportation that is not mandatory, contact with individuals with respiratory symptoms, and contact with high–risk people (i.e., vulnerable populations). Regarding face masks, only 26.5% and 19.8% of the sample wear face masks (always and very frequently, respectively) when they are in public settings. This is a percentage that one may consider exceptionally low. As to refraining from crowded places, this measure was adopted by participants, whereas 50.8% and 25.7% stated that they always and very frequently avoid crowded public places, while, with regard to all other practices, in the vast majority, citizens seem to comply with the proactive prevention tactics recommended by the government and World Health Organizations.

### 4.2. Addressing RQ2 (Objective No. 1) and RQ3 (Objective No. 2)

K–Means cluster analysis provided five meaningful segments of citizens’ behavior regarding COVID–19 (RQ2/objective No. 1), and chi–square tests contributed to developing each segment’s profile (RQ3/objective No. 2). This paper identified five segments of citizens based on their preventive behavior: The Meticulous Proactive Citizens, the Self–isolated Citizens, the Cautious Citizens, the Occasionally Cautious Citizens, and the Unconcerned Citizens. Previous studies that performed citizen segmentation based on prevention behavior toward COVID–19 do not exist, and, thus, we cannot make direct comparisons of findings. However, studies centered around behavior towards viruses and segmentation analysis mostly refer to sexual diseases (AIDS), or aspects of behavior (i.e., perceptions, knowledge, awareness) developed toward viruses and diseases, which are issues not tackled by this study. However, all studies [55,72] highlighted that, for effective public interventions, communication strategies must target each segment differently with regard to their attitudes and behavior [76].

### 4.3. Addressing RQ4 (Objective No. 3)

Communication strategies should be designed not only to inform but also to change perceptions and behaviors by aiming to manage the COVID–19 outbreak. Overall, with regard to the total sample of the current study, it seems a significant percentage of the participants follow the precautionary measures suggested. In order for communication to be more effective, it is crucial to plan and implement group–specific specialized communication strategies to accomplish proper dissemination of the messages conveyed [77].

More specifically, results revealed that the first segment, and largest group of the participants, respond well to the precaution measures. Members of the "Meticulous, proactive citizens" group include families and individuals from the vulnerable group above 65 years of age. It should be noticed that the findings are aligned with prior research, which indicates that higher risk perceptions can enhance positive behaviors [78]. Accordingly, the participants belonging to the third cluster of the "Cautious Citizens" frequently comply with the measures and follow the directions for protecting themselves and others. In addition, the second segment chose to be "Self-Isolated" instead of going to public places and, therefore, they opted for protecting themselves from COVID–19. Again, this segment is over–represented by female subjects, above 55 years old, and highly educated people. This result is aligned with previous research by Muto et al. [75], which claimed that females are more supportive of social distancing.

As far as targeted communication for the above segments, communication from official sources should continue to warn people against the imminent threats that might occur if individuals stop following the experts’ recommendations and start considering that the COVID–19 outbreak is over. Risk communication research indicates that advocating to take action for an imminent threat is more successful in motivating behavior adjustments [78,79]. Integrative marketing communication campaigns could be implemented with the dominant use of traditional media such as television, which is justified by the fact that an audience over 65 years of age is targeted in the first group. However, traditional media can also be combined with the use of digital (e.g., updated websites, articles) and social media, which may be effective in providing timely information during crisis periods [80] to better cover the second (and most educated) segment as well as the third segment. Both advertising and public relations should also be exercised by incorporating expert opinion and celebrity endorsement.

On the contrary, the fourth group that was identified in the study, consists mostly of males, up to 35 years old, with no family obligations who occasionally take preventive measures. Communication strategy for this group should incorporate the importance and effectiveness of the recommended proactive measures not only for personal protection but also for achieving a greater, societal result. Specially designed communication with an emphasis on digital media, and the strategic use of social media (e.g., hashtags) and social marketing interventions, should be implemented to members of the fourth group. 

Lastly, the fifth group seems to be comprised of very young individuals without children who seem not to take any measures to protect themselves. Because of their age, members of this group may feel that they face a rather low risk of complications from the disease and, therefore, they disregard the recommendations for changing behaviors [78]. As a result, communication that addresses the members of this least–complying group should not only indicate the benefits to the recipients from following the proactive measures, but also highlight the importance of protecting others, and the prospect of being approved by peers’ social groups. In all cases, messages need to be persuasive and infuse confidence in the effectiveness of proactive measures [81]. Digital campaigns, including the use of social media, should be implemented to target this cluster, which is the younger group. All forms of digital communication should be employed, e.g., social media campaigns, educational and creative videos on YouTube, and the formation of virtual groups and communities that support and promote the discussion around the critical need for taking the necessary actions for preventing the COVID–19 [80,81].

## 5. Conclusions, Limitations, and Directions for Future Research

The present study provided a framework of the degree to which Greek people followed the recommended measures for their protection from COVID–19. Segmentation analysis was performed on the basis of the proactive protective behavior of the individuals. Official sources should continue to provide clear guidance on how individuals should act in the current phase of the outbreak, even after the quarantine is over, in order to maintain the really successful results that were achieved. In order to keep long–term effectiveness, though, targeted communication must be put in force. This communication tactic can address the concerns of all different segments. It can create more appropriate and effective approaches for individuals with diverse backgrounds and behaviors toward the recommended measures for protecting against this disease. Clear phrasing and communication frequency are essential, together with credibility and certainty for the effectiveness of the proactive measures. Thus, it will be ensured that the population obtains accurate information and is protected from misleading sources, false data, and unreliable recommendations.

This research is not free of limitations, regardless of the authors’ attempt to reduce them. The most substantial limitation of this study is the implementation of a non–probability sampling method, which results in a lack of generalizability. Additionally, the sample of the research could be considered small compared to other studies. However, this research is self–funded, and, due to time and economic constraints, this sample could be collected under the specific conditions. The third limitation pertains to the variables used in the segmentation analysis, given that this study did not include psychographic variables, but was limited to citizens’ demographic and socioeconomic characteristics. Lastly, this work was limited to examining a single country (Greece). 

The above limitations could lead to future research directions by implementing a probability sampling frame with a larger sample, and incorporating citizens’ psychographic characteristics to validate the results of this study. Lastly, it would be of interest to replicate this research in other counties and compare and contrast citizens’ behavior.

## Figures and Tables

**Table 1:** Participants’ profile in the field research.

Sample Characteristics	Frequencies	Percentages (%)
Gender		
Male	1619	48.2
Female	1740	51.8
Age		
16–25	766	22.8
26–35	572	17.0
36–45	438	13.1
46–55	567	16.9
55–65	588	17.5
66+	428	12.7
Marital status		
Single	1373	40.9
Married	1597	47.5
Divorced	186	5.5
Widowed	203	6.1
Education		
Primary	434	12.9
Secondary	811	24.2
Postsecondary	478	14.2
Graduate	1331	39.6
Postgraduate	305	9.1
Profession		
Employee (public–private)	1244	37.0
Businessman	496	14.8
Labourer	84	2.5
Dependent (housekeeper, student, unemployed)	1041	31.0
Pensioner	494	14.7
Area of residence		
Urban	2002	59.6
Rural	1357	40.4
Net Monthly personal Income (€)		
≤350.00	724	21.6
350.01–1000.00	1685	50.1
1000.01–2000.00	751	22.3
2000.01+	199	6.0

Source: The authors.

**Table 2:** Citizens’ proactive behavior against COVID–19 (%).

Statements	1	2	3	4	5	6	7	MS
1. Avoid all non–mandatory transportation and travel	1.5	2.2	1.7	4.7	7.9	21.5	60.6	6.22
2. Avoid contact with individuals who have respiratory symptoms	1.5	1.8	2.4	4.0	9.3	21.3	59.7	6.21
3. Avoid contact with individuals at high risk for severe illness (vulnerable groups)	1.3	2.2	2.1	4.6	9.0	22.8	58.0	6.18
4. Use of hand sanitizer (that contains at least 60% alcohol) after touching objects and surfaces in public	2.6	1.6	1.9	3.5	9.3	22.5	58.7	6.17
5. Self–quarantine at home for 14 days following the last exposure with suspected infected individuals	2.0	2.6	2.2	4.4	9.4	18.6	60.8	6.16
6. Self–isolation for at least 14 days after contact with people who have come from abroad	2.3	2.3	2.2	4.6	7.5	20.6	60.5	6.16
7. Very good hand washing after touching objects and surfaces in public	1.5	2.3	2.9	4.5	9.9	22.5	56.4	6.12
8. Strict compliance with hygiene standards regarding shared toilets (evidence of fecal–oral transmission)	2.5	2.5	2.1	6.5	9.7	19.0	57.6	6.06
9. Strict adherence to hygiene rules at home	1.8	2.0	2.7	5.5	11.9	25.5	50.7	6.03
10. Obey the government restrictions to prevent COVID–19 disease	1.7	2.4	2.7	5.3	11.1	27.2	49.6	6.02
11. Avoid crowded and overcrowded public areas	2.0	2.5	2.6	5.2	11.2	25.7	50.8	6.01
12. Wash thoroughly fruits and vegetables	2.1	2.8	2.7	6.8	11.6	22.8	51.2	5.96
13. Limit transportation only to the necessary	2.1	2.5	3.5	6.0	12.7	25.3	48.1	5.93
14. Clean and disinfect objects and surfaces that are frequently touched by many people	2.1	2.6	2.7	6.8	12.8	25.1	47.8	5.92
15. Avoid touching the face (in particular, eyes, nose, and mouth) with hands	1.6	3.6	2.8	6.5	13.6	27.9	43.8	5.86
16. Always maintain a distance of at least 2 meters from others	1.6	2.9	3.2	7.0	15.3	25.9	44.1	5.86
17. Avoid contact with other people outside the immediate family environment as much as possible	2.1	2.4	3.4	6.8	15.6	26.6	43.2	5.84
18. Adopt respiratory hygiene (covering the cough or sneeze drops by wearing a face mask and washing my hands often.)	3.8	3.7	2.9	6.4	11.6	22.5	49.2	5.82
19. Social distancing by home isolation as much as possible	2.1	3.7	3.7	6.3	14.9	26.4	43.0	5.80
20. Regular update of the COVID–19 disease outbreak and precaution measures that should be implemented	3.0	3.4	3.8	7.4	14.9	25.8	41.8	5.72
21. Clean and disinfect packaged products	7.6	6.2	5.4	9.4	14.0	20.5	37.0	5.25
22. Movement to public services, organizations, and areas wearing hand gloves	9.3	5.7	5.5	7.8	13.2	22.3	36.2	5.22
23. Avoid contact with wild animals	9.4	6.6	5.1	11.6	11.6	17.6	38.1	5.15
24. Wear single–use hand gloves in public settings	10.5	6.0	5.4	8.7	14.1	20.2	35.0	5.11
25. Use a mask when using public transportation	14.5	6.0	6.2	8.5	13.7	19.2	32.0	4.86
26. Use a mask when in public	15.3	7.1	5.9	10.0	15.4	19.8	26.5	4.69
27. Check daily body temperature, monitoring for fever, cough, or dyspnea	19.2	10.8	8.9	13.5	14.2	15.1	18.2	4.11

Source: the authors.

**Table 3:** Segments based on participants’ proactive protective behavior.

Preventive Actions	Cluster/Segments					ANOVA
	1st *N* = 1634	2nd *N* = 599	3rd *N* = 636	4th *N* = 338	5th *N* = 152	F
	Meticulous Proactive	Self–Isolated	Cautious	Occasionally Cautious	Unconcerned	
1. Use of hand sanitizer (containing at least 60% alcohol) after touching objects and surfaces in public	6.8	6.7	5.8	4.8	2.4	1136.103
2. Avoid touching the face (in particular eyes, nose, and mouth) with hands	6.6	6.0	5.5	4.3	2.4	912.566
3. Avoid crowded and overcrowded public areas	6.7	6.4	5.6	4.3	2.5	1140.336
4. Clean and disinfect objects and surfaces that are frequently touched by many people	6.6	6.1	5.6	4.3	2.3	1001.522
5. Avoid contact with individuals who have respiratory symptoms	6.8	6.6	5.8	5.0	2.5	1135.288
6. Avoid contact with other people outside the immediate family environment as much as possible	6.6	6.0	5.4	4.2	2.2	1158.045
7. Social distancing by home isolation as much as possible	6.6	6.1	5.3	3.9	2.3	1134.638
8. Avoid contact with wild animals	5.9	5.2	4.5	3.8	2.3	235.960
9. Always maintain a distance of at least 2 meters from others	6.6	6.2	5.4	4.1	2.3	1444.547
10. Very good handwashing after touching objects and surfaces in public	6.8	6.6	5.7	4.7	2.2	1485.717
11. Use a mask when in public	6.1	2.0	5.0	3.1	2.2	1276.645
12. Strict compliance with hygiene standards regarding shared toilets (evidence of fecal–oral transmission)	6.7	6.5	5.6	4.7	2.2	890.936
13. Self–quarantine at home for 14 days following the last exposure with suspected infected individuals	6.8	6.7	5.8	4.7	2.3	1241.756
14. Avoid all non–mandatory transportation and travel	6.8	6.8	5.8	5.1	2.5	1239.797
15. Self–isolation for at least 14 days after contact with people who have come from abroad	6.8	6.6	5.8	4.7	2.3	1177.999
16. Strict adherence to hygiene rules at home	6.7	6.4	5.5	4.7	2.1	1471.019
17. Avoid contact with individuals with high risk for severe illness (vulnerable groups)	6.8	6.6	5.7	4.9	2.4	1549.261
18. Wear single–use hand gloves in public settings	6.5	3.0	5.3	3.1	2.1	1474.820
19. Clean and disinfect packaged products	6.4	4.1	5.1	3.5	2.3	647.507
20. Limit transportation only to the necessary	6.7	6.2	5.5	4.0	2.4	1363.492
21. Movement to public services, organizations, and areas wearing hand gloves	6.5	3.3	5.5	3.1	2.2	1335.615
22. Use a mask when using public transportation	6.4	2.0	5.3	3.0	2.2	1954.202
23. Wash fruits and vegetables thoroughly	6.7	6.3	5.5	4.4	2.2	1116.495
24. Regular update of the COVID–19 disease outbreak and precaution measures that should be implemented	6.5	5.8	5.3	4.1	2.4	702.551
25. Adopt respiratory hygiene (covering the cough or sneeze drops by wearing a face mask and washing my hands often.)	6.7	5.3	5.7	4.4	2.2	697.239
26. Obey the government restrictions to prevent COVID–19 disease	6.7	6.3	5.7	4.3	2.3	1464.415
27. Check daily body temperature, monitoring for fever, cough, or dyspnea	5.2	2.4	4.1	2.8	2.1	396.910

Source: the authors.

**Table 4:** Chi–square results between citizens’ socioeconomic and demographic characteristics and clusters.

Socioeconomic/Demographic Characteristic	Pearson ×2	df	*p*-Value
Gender	67.230	4	0.000
Age	98.902	24	0.000
Marital status	67.933	12	0.000
Education	103.456	20	0.000
Profession	85.869	20	0.000
Existence of children <18 years old in the family	17.595	4	0.002
Area of residence (rural or urban)	11.697	4	0.021
Personal income	56.260	16	0.000

Source: The authors.

**Table 5:** Segments’ profile.

Socioeconomic and Demographic Characteristic	Cluster/Segment				
	1st	2nd	3rd	4th	5th
	Meticulous Proactive	Self-Isolated	Cautious	Occasionally Cautious	Unconcerned
Gender					
Male	44.4	40.7	55.3	60.7	61.2
Female	55.2	59.3	44.7	39.3	38.8
Age					
<25	20.2	17.5	20.8	26.3	37.9
26–35	14.6	20.2	17.1	21.3	20.4
36–45	13.5	12.4	12.6	13.0	13.2
46–55	16.7	18.5	18.9	16.0	5.9
55–65	19.5	22.7	18.6	13.6	14.7
66 +–	15.5	8.7	12.1	9.7	7.9
Marital status	1	5	3	2	4
Single	35.7	44.1	39.2	50.9	52.6
Married	50.1	43.4	51.1	38.8	41.4
Divorced	7.0	8.3	4.7	3.8	2.0
Widowed	7.2	4.2	5.0	6.5	3.9
Children (<18) in family					
Yes	39.3	34.9	32.7	30.9	28.5
No	60.7	65.1	67.3	69.1	71.5
Education					
Has not finished elementary school	3.9	0.8	2.0	3.3	12.5
Primary	11.9	5.3	8.8	9.5	5.9
Secondary	23.9	21.0	27.2	26.9	25.4
Postsecondary	14.2	13.7	14.2	15.4	14.5
Graduate	37.9	47.2	39.8	35.8	36.2
Postgraduate	8.3	11.9	8.0	9.2	5.5
Profession					
Public employee	14.0	16.7	12.3	10.7	11.2
Private employee	24.4	22.5	23.7	20.4	21.1
Businessman	13.4	16.0	15.1	18.9	13.8
Laborer	1.7	1.5	3.5	4.4	6.6
Pensioner	17.6	9.8	16.7	9.2	7.2
Dependent (housekeeper, student, unemployed)	29.0	33.4	28.8	36.4	40.1
Area of residence					
Urban	59.2	64.9	59.1	55.9	53.3
Rural	40.8	35.1	40.9	44.1	46.7
Net monthly personal income (€)					
≤350.00	20.4	25.9	17.9	27.2	19.1
350.01–600.00	22.6	24.5	26.7	21.9	16.4
600.01–1000.00	29.0	21.2	26.1	27.2	26.3
1000.01–2000.00	22.5	22.5	23.9	18.0	23.7
2000.01+	5.4	5.8	5.3	5.6	14.5

Source: the authors.
